# Simulation of Vegetation Carbon Sink of Arbor Forest and Carbon Mitigation of Forestry Bioenergy in China

**DOI:** 10.3390/ijerph192013507

**Published:** 2022-10-19

**Authors:** Xiaozhe Ma, Leying Wu, Yongbin Zhu, Jing Wu, Yaochen Qin

**Affiliations:** 1 College of Geography and Environmental Science, Henan University, Kaifeng 475004, China; 2 Key Laboratory of Geospatial Technology for the Middle and Lower Yellow River Regions (Henan University), Ministry of Education, Kaifeng 475004, China; 3 Key Research Institute of Yellow River Civilization and Sustainable Development & Collaborative Innovation Center on Yellow River Civilization, Henan University, Kaifeng 475001, China; 4 Regional Planning and Development Center, Henan University, Kaifeng 475004, China; 5 Institutes of Science and Development, Chinese Academy of Sciences, Beijing 100190, China

**Keywords:** arbor forest, vegetation carbon sink, bioenergy, CO2FIX model

## Abstract

Mitigating carbon emissions through forest carbon sinks is one of the nature-based solutions to global warming. Forest ecosystems play a role as a carbon sink and an important source of bioenergy. China’s forest ecosystems have significantly contributed to mitigating carbon emissions. However, there are relatively limited quantitative studies on the carbon mitigation effects of forestry bioenergy in China, so this paper simulated the carbon sequestration of Chinese arbor forest vegetation from 2018 to 2060 based on the CO2FIX model and accounted for the carbon emission reduction brought about by substituting forestry bioenergy for fossil energy, which is important for the formulation of policies to tackle climate change in the Chinese forestry sector. The simulation results showed that the carbon storage of all arbor forest vegetation in China increased year by year from 2018 to 2060, and, overall, it behaved as a carbon sink, with the annual carbon sink fluctuating in the region of 250 MtC/a. For commercial forests that already existed in 2018, the emission reduction effected by substituting forestry bioenergy for fossil energy was significant. The average annual carbon reduction in terms of bioenergy by using traditional and improved stoves reached 36.1 and 69.3 MtC/a, respectively. Overall, for China’s existing arbor forests, especially commercial forests, forestry bioenergy should be utilized more efficiently to further exploit its emission reduction potential. For future newly planted forests in China, new afforestation should focus on ecological public welfare forests, which are more beneficial as carbon sinks.

## 1. Introduction

China’s “Carbon Peaking and Carbon Neutrality” target is an important pledge to mitigate carbon emissions and improve the ecological environment [1,2,3]. Among various emission reduction measures to achieve this goal, nature-based solutions, such as carbon sequestration in forests, have attracted a lot of attention from society [4,5,6]. As a major component of the terrestrial biosphere, forest ecosystems play an important role in regulating the climate, carbon cycling, and mitigating climate warming [7,8,9]. Approximately 50% of organic carbon in the terrestrial biosphere can be stored by forests, and global forests can increase carbon dioxide sequestration by 32% [10]. The carbon sinks of China’s forest land contributed more than 90% of the country’s total carbon sinks from land use between 1999 and 2014 [11]. Therefore, enhancing forest carbon sinks is considered to be an important means of reducing carbon emissions, and has become an essential strategy recognized by the international community to mitigate climate change [12,13,14].

Data from the ninth China forest resources inventory show that China’s forest area reached 220 Mha in 2018, with a forest coverage rate of 22.96%. Among them, China’s planted forests cover an area of 80 Mha, making China the world’s fastest growing country in terms of planted forest area. As such, China has become a leading force in global greening, and its forest ecosystems as a whole behave like a carbon sink, playing an important role in mitigating carbon emissions [7,15,16,17,18]. Regarding research on China’s forest carbon sink, although there are some differences in research perspectives and methods, simulation results generally indicate that China’s forests are huge carbon sinks [19,20,21]. Sun and Liu historically reviewed select studies on China’s forest carbon sequestration and pointed out that the carbon storage of China’s forest ecosystem is 28.90 PgC, of which vegetation carbon sequestration is 8.65 ± 1.52 PgC (after 2007), soil carbon storage reaches 21.16 ± 5.39 PgC, and litter carbon storage is only 0.86 ± 0.08 PgC [15]. From 1981 to 2000, 14.6–16.1% of CO_2_ emissions from fossil fuel combustion in China were absorbed by forest vegetation carbon sinks [22,23]. Scientists are also optimistic about the future carbon sequestration of China’s forests, and generally believe that it will continue to exhibit an increasing trend in the following years [24]. Yao et al. pointed out that the total carbon sequestered by Chinese forest biomass during the 2000s was estimated to be 10.75 ± 0.005 PgC, with a mean carbon density of 71.9 MgC/ha. Chinese total forest biomass would increase by 8.89–10.37 PgC by the end of the 2040s [20]. Qiu et al. showed that the carbon stock, density, and sink of forest vegetation in China exhibit a significant increasing trend from 2003 to 2050. The cumulative carbon sink of Chinese forest vegetation from 2020 to 2050 would be 5.52 PgC, which is about 2.2 times the total carbon sink from 2000 to 2020 and is the equivalent of 22.14% of the accumulated carbon emissions from fossil fuels in China from 2020 to 2050 [23]. In addition, China’s National Forestry and Grassland Administration has proposed that China should further strengthen new afforestation and enhance the total amount of forests from 2016 to 2050. By 2050, the national forest coverage rate will be stabilized at over 26% and the total carbon stock of forest vegetation will reach over 13 PgC [25]. This implies that China’s forest ecosystems have great potential for carbon sequestration in the future and will contribute significantly to the mitigation of greenhouse gas emissions, especially from newly planted forests. Although the above studies have done a decent analysis of China’s forest carbon sinks, unfortunately, these studies did not analyze the effects of forest species on forest carbon stock, nor did they consider the emission reduction effects of forestry resource reuse, such as the use of forestry bioenergy, which makes the above research on forest carbon sequestration less comprehensive and in need of further improvement.

In fact, forest ecosystems are not only an important carbon sink but also one of the most important sources of bioenergy [26,27]. Bioenergy is considered to be another important energy source after coal, oil, and natural gas [28]. From the perspective of carbon emissions, bioenergy is generally considered to be a perfect substitute for fossil energy which can effectively reduce carbon emissions caused by fossil fuels [29,30]. In addition, it offers other advantages such as strengthening energy security and enhancing ecological services [28,31,32,33]. Forests are one of the main sources of bioenergy, and the litter from the growth of forests or the residues from the processing of forest products can be converted into bioenergy [34]. In contrast to fossil energy sources, the burning of bioenergy only temporarily releases carbon sequestered in forest biomass into the atmosphere, and as the plants grow again, the previously released carbon is reabsorbed and sequestered. If the cycle of growth and harvest is sustained, there is no net release of carbon. In other words, forestry bioenergy can be carbon neutral [29]. Compared with other types of bioenergy, forestry bioenergy presents many advantages. It can avoid the competition for land as well as food production problems faced by crop bioenergy and has a continuous ecological service function [33,35]. China offers a wealth of forestry biomass resources, and the production of forest residues in China is estimated to rise from 2.2 Mt in 2007 to 2.6 Mt in 2016 [35,36]. This bioenergy serves as a resource not only for rural heating and firewood but also for electricity generation [32]. For a low-carbon future, the quantitative and qualitative assessment of the carbon reduction potential of forestry bioenergy is important in the context of China’s aim to achieve the “Carbon Peaking and Carbon Neutrality” target. However, current research on forestry bioenergy is relatively limited and needs to be further strengthened.

Based on a large body of studies on the assessment of forest carbon sinks, it can be found that the current methods for measuring carbon sinks can be roughly classified into three categories: forest inventories, satellite remote sensing, and process-based simulation [15,23,37,38,39]. Although these methods are applied to estimate forest carbon sinks at different temporal and spatial scales, they have their advantages and limitations. The process-based model is an important method in forest carbon sink research. It mainly employs models to complete the simulation of carbon sink potential, which, to a certain extent, makes up for the deficiencies of forest inventories and remote sensing estimation [38,40]. Most process-based models focus on the carbon cycle within forest ecosystems, and fewer involve the carbon retained in forest products and forestry bioenergy in their forest carbon cycle analysis [41]. In contrast, the CO2FIX model is a typical process-based model whose main feature is the incorporation of forest products and bioenergy modules into the model, providing a more integrated carbon cycle chain of forest ecosystems [39,42,43,44]. This model can effectively simulate the carbon stock of forest ecosystems and the emission reduction potential of forestry bioenergy, and so is widely applied in the study of the forest carbon cycle and forestry biomass [44,45,46]. Therefore, this paper used the CO2FIX model, combined with data from the ninth China forest resources inventory, to simulate and predict the carbon sequestration of China’s arbor forest vegetation from 2018 to 2060. This paper further incorporated the role of forestry bioenergy as a substitute for coal and analyzed the carbon reduction in terms of arbor forest bioenergy, which is important in the context of the Chinese forestry sector’s attempts to formulate reasonable policies to cope with climate change.

## 2. Materials and Methods

### 2.1. Methodology

The carbon density of forest vegetation directly affects its carbon sink, so the estimation of carbon density is one of the key issues in forest carbon sink simulation. In this paper, the simulation of the carbon density of Chinese arbor forest vegetation was accomplished by using the CO2FIX model. This model, which originated from Wageningen University in the Netherlands, utilizes the biomass-storage equation to simulate the carbon cycle of forest succession with annual time units. The CO2FIX model mainly includes biomass, soils, forest products, bioenergy, economic modules, and carbon accounting modules [42]. The biomass, soil, and forest products modules are used to simulate the carbon density of forest vegetation, while the bioenergy module is designed to analyze the carbon reduction effect of forestry bioenergy [29]. Based on this model, it is feasible to simulate the carbon density of vegetation for each tree species at each stand age. Combining the carbon density data with the area data of tree species, the carbon storage and annual carbon sink of forest vegetation can be obtained, as shown in Equations (1) and (2):

*C_i_*_,*t*_ = *D_i_*_,*t*_ · *A_i_*_,*t*_ (1)

*S_i_*_,*t*_ = *C_i_*_,*t*_ − *C_i_*_,*t*−1_ (2)

where *C*_*i*,*t*_ is the vegetation carbon storage of tree species *i* in the year *t*, *D*_*i*,*t*_ is the vegetation carbon density of tree species *i* in the year *t*, *A*_*i*,*t*_ is the area of tree species *i* in year *t*, and *S*_*i*,*t*_ is the vegetation carbon sink of tree species *i* in the year *t*.

Forestry bioenergy can be carbon neutral [29]. Therefore, replacing fossil energy with forestry bioenergy provides an opportunity to abate carbon emissions. This paper assessed the effectiveness of this abatement using the bioenergy module in the CO2FIX model. The bioenergy module provides two ways to reduce carbon emissions, either by replacing fossil energy with bioenergy or by improving energy combustion techniques to increase energy utilization efficiency [29]. Hence, the scale of carbon abatement is mainly affected by the heat value of bioenergy and fossil energy, the efficiency of energy utilization, and the greenhouse gas emission factors of the technologies being substituted and their alternatives. The greenhouse gas emission factors vary between energy sources and between the substituted and alternative technologies, so the amount of greenhouse gas emissions released by the same heat generation can be different. The difference in emissions is the carbon emission reduction achieved by replacing fossil energy with bioenergy. The specific formulation is as shown in Equations (3)–(5) [29].

*GHGmit_j_* = *E_sj_* − *E_aj_* (3)

where *GHGmit_j_* is the emission mitigation in terms of greenhouse gas, which includes CO_2_, CH_4_, N_2_O, CO, and TNMOC. *E_sj_* is the amount of greenhouse gas *j* released by the fossil fuel or technology to be substituted. *E_aj_* is the amount of greenhouse gas *j* released by an alternative technology. The emissions of the alternative technology can be calculated according to:

*E_aj_* = *FI* · *ef_aj_* (4)

where *FI* is the bioenergy input and *ef_aj_* is the emission factor for the alternative technology for each greenhouse gas *j*. The equivalent emission of fossil fuels or the technology to be substituted is calculated according to:

*E_sj_* = *FI*·(*EC_a_*/*EC_s_*)·(*ŋ_a_*/*ŋ_s_*)·*ef_s_*_j_ (5)

where *EC_a_* is energy content of the alternative fuel (bioenergy), *EC_s_* is the energy content of the replaced fossil fuel, *ŋ_a_* is the energy efficiency of the alternative technology, *ŋ_s_* is the energy efficiency of the technology to be substituted, and *ef_sj_* is the emission factor of the fuel/technology to be substituted for each greenhouse gas *j*.

### 2.2. Parameters and Data Sources

The data used in this paper are mainly from the latest forest inventory data in China, i.e., the ninth China forest resources inventory. The statistical year of the inventory data ended in 2018, so the initial year of the simulation was set to 2018. In addition, considering China’s major strategic decision to achieve carbon peaking and carbon neutrality, the end year of the simulation was set to 2060. Therefore, this paper employed the CO2FIX model to simulate the carbon sequestration of Chinese arbor forest vegetation from 2018 to 2060 based on dominant tree species. The tree species to be modelled is the primary thing that needs to be specified in this paper. It is well known that the carbon storage of forest vegetation is directly related to the stock volume. Thus, according to the data of the ninth China forest resources inventory [47], this paper selected 19 dominant tree species with relatively large stock volumes and areas as the simulation objects, ignoring some tree species with limited stocks and areas, as shown in Table 1. The total volume of the 19 dominant tree species reached 15,971.5 Mm^3^, accounting for 93.6% of the national total volume of arbor forests, and the total area reached 161.8 Mha, accounting for 89.9% of the national total arbor forest area. Therefore, the carbon sink simulation of these 19 dominant tree species can fully illustrate the current status and evolutionary trend of carbon sequestration in China’s arboreal forest vegetation.

Once the dominant tree species to be modelled are identified, the area for each species needs to be clarified. In this paper, we not only simulated the vegetation carbon sink of arbor forests already planted in China at the time of the ninth China forest resources inventory but also predicted the carbon sink of China’s newly planted forests in the future. So, we divided the carbon sink simulation into two parts, i.e., the existing forest and the new afforestation. For the existing arbor forests, the area data for each dominant species were obtained from the ninth China forest resources inventory. Considering the differences in vegetation carbon sequestration among forest species, this paper further divided each tree species into two types: public welfare forest and commercial forest. Public welfare forests mainly included shelter forests and special-purpose forests, which are used to undertake ecological functions. This paper did not consider the impact of deforestation on its carbon storage. Commercial forests mainly involved timber forests, charcoal forests, and economic forests. This paper assumed that there was a logging cycle for commercial forests, so their carbon stocks were directly affected by deforestation. The area data for public welfare forests and commercial forests of various tree species came from the ninth China forest resources inventory [47], as shown in Table 1.

For new afforestation, the first thing that needed to be clarified was the area of the newly planted forest. China’s existing forest area has reached 220.5 Mha, with a forest coverage rate of 22.96%, including 179.9 Mha of arbor forests. According to the national forest plan, China’s forest coverage rate will be 26% in 2035 and will rise to the world average of 30.7% by 2050 [47]. As a result, China’s forest area would increase to 249.6 Mha in 2035 and 294.8 Mha in 2050. This paper assumed that all new plantations would be arbor forests. The newly planted area during the afforestation period was equally distributed for all years, i.e., 1.7 Mha per year from 2019 to 2035 and 3.0 Mha per year from 2036 to 2050. The annual afforestation area was allocated to each tree species according to the proportion of its area to the total arbor forest area in 2018 [23], so the total area of arbor forests in China was estimated to reach 254.2 Mha by 2050.

In addition to area data for each tree species, carbon density data are also required for the simulation of forest vegetation carbon sinks in China. This paper employed the CO2FIX model to simulate the carbon density of each dominant tree species for each year. The biomass module of this model needed to set the wood density of tree species, the current annual increment of branches and leaves, the mortality rate, the growth period, and the turnover rate. These parameters refer to the settings of Ma and Wang [17]. In particular, it should be noted that carbon content is one of the key parameters in estimating forest carbon stocks. The carbon content in the CO2FIX is set by default to 0.5 as provided by the IPCC. In fact, the carbon content varies greatly between species and organs due to a number of factors such as location and season. Using 0.5 as the carbon content factor would result in a 10% bias in carbon stock [48]. Therefore, based on the study of Wang et al. [48], the carbon contents of the different organs of 19 dominant tree species were updated in this paper, as shown in Table 2. Furthermore, the simulation of the carbon density of commercial forests requires the parameters of thinning and final felling in the biomass module. This paper referred to Ma and Wang and the National public service platform for standards information for these settings [17,49].

In the CO2FIX soil module, the monthly mean temperature and mean precipitation data were taken from the World Climate website (http://www.worldclimate.com/). It is worth noting that this paper modelled the carbon density of tree species on a national scale. However, there are significant local geographical characteristics regarding tree growth, so the downscaling of climate data is a concern in this paper. To solve this problem, this study first selected the main provinces where a certain tree species was distributed based on the data of the ninth China forest resources inventory and then calculated the species’ volume of unit area in the whole nation and each province. Then, this study chose the province with the closest volume of unit area to the national level, and finally the average climate data of multiple cities in this province was employed as the climate data of the soil module. The remaining parameters applied the default values of the CO2FIX model.

The bioenergy module of the CO2FIX model is mainly used to evaluate the emission reduction in terms of forestry bioenergy as an alternative to fossil energy. It is assumed that commercial forest has a harvesting cycle, and its logging residues and wastes during wood product processing can be used as bioenergy [32,50]. So, this paper analyzed the emission reduction effect of substituting bioenergy for coal through this module. The reason for replacing coal with bioenergy is that coal is commonly used for heating or cooking in rural or peri-urban areas, where forest residues and waste from forest products are more easily available [51]. Thus, this paper argues that replacing coal with bioenergy is more reasonable in rural areas of China. The combustion technology of bioenergy has a relatively large impact on its carbon reduction effect. So, two technologies were provided in this paper, traditional cookstoves and improved stoves, as shown in Table 3. This paper simulated the emission reduction as a result of bioenergy using these two technologies separately. The bioenergy module needs parameters such as the greenhouse gas emission coefficient, fuel calorific value, and utilization efficiency of bioenergy and coal to be provided, among which parameters such as calorific value and utilization efficiency adopt the default values of the model. The greenhouse gas emission coefficients of each energy utilization technology were provided by the CO2FIX model and IPCC guidelines for national greenhouse gas inventory, as shown in Table 3.

## 3. Results

### 3.1. Vegetation Carbon Sink of Tree Species

**Figure 1:**
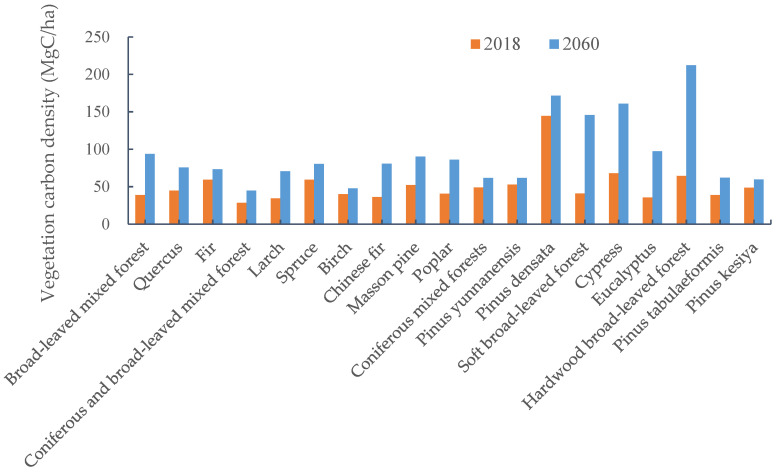
Vegetation carbon density of dominant tree species in China’s existing arbor forests.

In this paper, the arbor forests that already existed at the time of the ninth China forest resources inventory were set as existing arbor forests. The simulation results for the vegetation carbon density of dominant tree species in existing tree forests are shown in Figure 1. The vegetation carbon density of each dominant tree species in 2018 varied widely, between 28.4 MgC/ha and 144.3 MgC/ha. At the beginning of the simulation, the vegetation carbon densities of *pinus densata*, cypress, fir, spruce, and hardwood broad-leaved forests were relatively large, while coniferous and broad-leaved mixed forest, larch, *eucalyptus* were relatively small. With the annual growth of trees, the vegetation carbon density of all tree species had significantly improved by 2060, ranging between 44.6 MgC/ha and 212.2 MgC/ha. The vegetation carbon density of *pinus densata*, cypress, and hardwood broad-leaved forest remained high in 2060, while that of coniferous and broad-leaved mixed forest, birch was relatively low. The vegetation carbon density of soft broad-leaved forest, hardwood broad-leaved forest, *eucalyptus*, broad-leaved mixed forest, and cypress increased dramatically during the simulation period, since these species were dominated by young and middle-aged forests at the beginning of the simulation. In the case of hardwood broad-leaved forest, *eucalyptus*, and cypress, the sum of their young and middle-aged forests accounted for nearly 80% of the total area of the species, while the sum of the young and middle-aged forests of soft broad-leaved forest accounted for 58.3%. During the simulation period, the forests of these tree species grew faster and gradually matured, so their vegetation carbon density grew rapidly. Fir, spruce, birch, *pinus yunnanensis*, and *pinus densata* had a larger proportion of mature forests during the simulation period, so the growth rate of these species was relatively low. In addition, the forests of these tree species were mainly public welfare forests, and deforestation has less of an impact on them, so the growth of vegetation carbon density of these tree species was limited.

**Figure 2:**
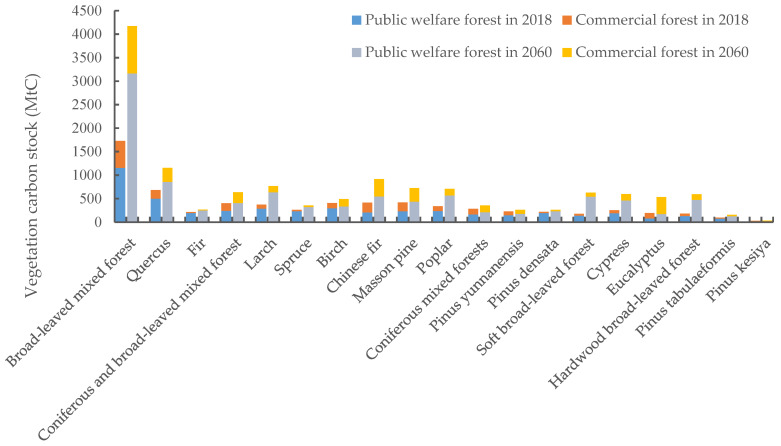
Vegetation carbon stocks of dominant tree species in China’s existing arbor forests.

The vegetation carbon storage of the 19 dominant tree species in the existing arbor forest from 2018 to 2060 is shown in Figure 2. The total carbon storage of the 19 dominant tree species increased by 97.6% during the simulation period, from 6876.9 MtC in 2018 to 13,591.6 MtC in 2060, with an average annual growth rate of 1.64%. The relatively large carbon stocks of broad-leaved mixed forest and quercus were directly related to their large distribution areas. These two species accounted for 24.8% and 8.5% of the national arbor forest area in 2018, respectively, making them the two largest tree species of China’s arbor forests in terms of area. The carbon storages of hardwood broad-leaved forest, soft broad-leaved forest, and *eucalyptus* significantly improved throughout the simulation period, mainly because their vegetation carbon density rose remarkably in the latter years of the simulation. As shown in Figure 2, most of the existing dominant tree species were dominated by public welfare forests, so most of the carbon stocks of forest species derived from public welfare forests. During the modelling period, the areas of public welfare forests made up of fir, spruce, and *pinus densata* were much larger than that of their commercial forests, and therefore these species had a higher share of public welfare forest carbon stocks, close to 90%. Unlike most tree species, *eucalyptus* forest is predominantly commercial forests, which covered an area of 362.2 10^2^ ha in 2018, approximately twice the area of public welfare forests. Despite the cyclical logging of *eucalyptus* commercial forests, these forests have a rapid growth rate and a relatively short growth cycle. The continuous deforestation kept *eucalyptus* forests in a state of rapid growth overall, so that *eucalyptus* carbon stocks were dominated by commercial forests, which accounted for 60.6% and 68.1% of carbon stocks in 2018 and 2060, respectively.

In order to depict the carbon sinks of each dominant tree species more clearly, the cumulative carbon sink of the 19 dominant tree species from 2019 to 2060 is shown in Table 4. The total cumulative carbon sequestered by the 19 dominant tree species during the study period was 6714.7 MtC, of which 5215.6 MtC was fixed by public welfare forests and 1499.1 MtC by commercial forests. The main sources of carbon sinks in public welfare forests were broad-leaved mixed forest, quercus, soft broad-leaved forest, and hardwood broad-leaved forest, while the main sources of carbon sinks in commercial forests were *eucalyptus*, Chinese fir, and masson pine. Over the study period, the cumulative carbon sinks of broad-leaved mixed forest and *eucalyptus* were relatively high, up to 2445.8 MtC and 1014.4 MtC, respectively. It should be noted that the cumulative carbon stock from commercial forests of fir was negative. This is because the vegetation carbon stock of fir significantly reduced in the latter years of the simulation due to human harvesting, which was lower than that in the early part of the simulation.

### 3.2. Vegetation Carbon Sink of All Arbor Forests

In this paper, the simulation of the vegetation carbon sequestration of China’s arbor forests was divided into two parts, with one part being the carbon sequestration of the existing arbor forest and the other being the carbon sequestration of new afforestation. It should be stressed that the existing arbor forests were those that already existed in China at the time of the ninth China forest resources inventory.

**Figure 3:**
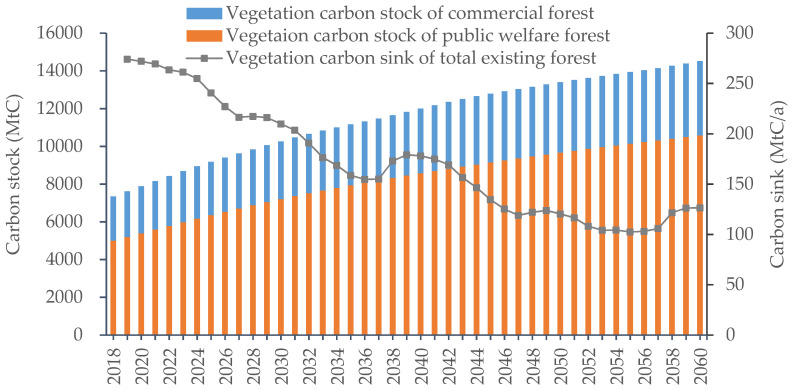
Vegetation carbon sink of China’s existing arbor forest.

The vegetation carbon storage of existing arbor forests in China grew steadily during the modelling period, with an average annual growth rate of 1.64%, as shown in Figure 3. In 2018, the vegetation carbon storage of China’s existing arbor forests was 7344.8 MtC, which is close to the 7575.4 MtC published in the ninth China forest resources inventory [47]. The vegetation carbon storage of public welfare forests was 5004.8 MtC, accounting for roughly 68.1% of existing arbor forests, and that of commercial forests was 2340.0 MtC, accounting for roughly 31.9%. After 2018, the carbon stock of existing arbor forests in China grew at a slow pace, and it reached 10,267.4 MtC by 2030. By the end of the simulation, China’s existing arboreal vegetation carbon stock reached 14,516.4 MtC, a 97.6% increase from 2018, mainly due to the dramatic increase in the carbon stock of public welfare forests. In 2060, China’s existing vegetation carbon stocks in public welfare forests rose to 10,575.3 MtC, doubling from 2018, while carbon stocks in commercial forests rose to 3941.7 MtC, an increase of 68.4% from 2018. The share of vegetation carbon stocks of public welfare forests in existing forests increased slightly to 72.6% by 2060, while the share of commercial forests decreased to 27.4%. Along with a rise in carbon stocks, the carbon density of existing tree forest vegetation in China also displayed an upward trend, increasing from 40.8 MgC/ha in 2018 to 80.7 MgC/ha in 2060.

The cumulative carbon sequestered by existing tree forest vegetation in China from 2019 to 2060 was 7171.5 MtC, with an annual average of 170.8 MtC/a, of which 132.6 MtC/a was derived from public welfare forests and 38.1 MtC/a from commercial forests. Overall, the annual carbon sink of total existing arbor forest presented a decreasing trend, as shown in Figure 3. It dropped from 274.2 MtC/a in 2019 to 126.4 MtC/a in 2060, which is due to the age of existing arbor forests in China. Data from the ninth China forest resources inventory indicated that China’s existing arbor forests were dominated by young and middle-aged forests in 2018, accounting for 63.9% of the total area [47]. Additionally, the existing arbor forests in China are mainly public welfare forests. With continuous growth in terms of tree age, most of the existing public welfare forests tended to mature naturally, and their carbon sequestration capacity was lower than that of young and middle-aged forests. Therefore, the annual carbon sink of existing arbor forests in China decreased. Figure 3 also shows that the annual carbon sink of arbor forests fluctuated throughout the entire study period, which was due to cyclical deforestation. The carbon sink capacity of commercial forests decreased significantly after the start of logging and gradually increased after the end of logging. Therefore, the annual carbon sink of the entire arbor forest showed regular fluctuations during the simulation period.

The above was a description of vegetation carbon sinks in China’s existing arbor forest. Next, the simulation results for carbon sequestration in new afforestation were illustrated. According to the national forest plan [47], China’s forest coverage rate would increase to the world average of 30.7% by 2050. Based on this plan, this paper simulated the carbon stock of new afforestation from 2019 to 2060. Considering the impact of forest species on vegetation carbon sequestration, two forest species were used for new plantations, either as public welfare forests or as commercial forests.

**Figure 4:**
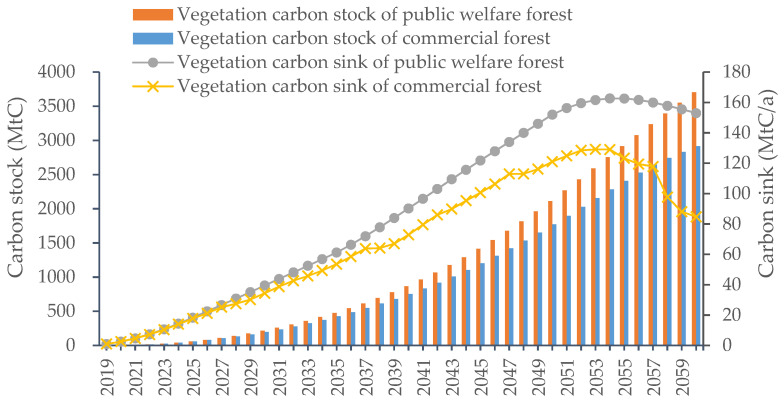
Vegetation carbon sink by new afforestation.

Figure 4 shows the carbon sequestration of new afforestation in China during the simulation period. When all newly planted forests were used as public welfare forests, their carbon storage increased from 0.9 MtC in 2019 to 3702.8 MtC in 2060, with this being directly related to the yearly increase in the new afforestation area. In 2019, China had 1.7 Mha of new plantations, and by 2050, the cumulative area of new plantations reached 7.4 Mha. When all new plantations were public welfare forests, their annual vegetation carbon sink rose significantly from 0.9 MtC/a in 2019 to 162.7 MtC/a in 2054 owning to the increasing afforestation area. At the latter stage of the simulation, the afforestation area did not increase, and the carbon sink of newly planted public welfare forests decreased to 152.9 MtC/a in 2060. This decline was mainly because of the maturity of tree species. From 2019 to 2060, the cumulative carbon sequestered by new public welfare forests was 3702.8 MtC, and its vegetation carbon density increased year by year, reaching 49.8 MgC/ha by 2060. When deforestation was considered and all newly planted forests were commercial forests, the carbon stock went up every year during the simulation period, as shown in Figure 4. The carbon stock of newly planted commercial forests reached 2923.56 MtC by 2060, which is lower than that of newly planted public welfare forests. The reason for this is that periodic anthropogenic harvesting had an influence on the new commercial forests’ carbon sink and led to a lower carbon sink than that of new public welfare forests. The peak carbon sink of new commercial forests in 2054 reached 129.0 MtC/a. During the modelling period, the cumulative carbon sequestration of new commercial forests was 2914.3 MtC, and the vegetation carbon density reached 39.2 MgC/ha by 2060. Overall, the carbon sequestration capacity of newly planted public welfare forests was greater than that of commercial forests, mainly because there was no anthropogenic deforestation in public welfare forests.

Combining the carbon sequestration data of existing arbor forests and new afforestation, it can be found that the annual carbon sequestration of total arbor forests in China from 2019 to 2060 fluctuated around 250 MtC/a due to periodic logging. Despite the fluctuations, China’s arbor forests as a whole performed as a carbon sink. As shown in Table 5, according to China’s forest resource planning [47], the area of arbor forests reached 254.2 Mha by 2050. When all new afforestation was used as public welfare forests, China’s total arbor forest vegetation absorbed an average of 258.9 MtC/a of carbon per year during the simulation period, with cumulative carbon sequestration of 10,874.3 MtC. In this scenario, the vegetation carbon density of China’s arbor forests was 40.8 MgC/ha in 2018, rising to 71.7 MgC/ha by 2060, close to the global average carbon density of forest vegetation of 71.6 MgC/ha [15]. When new afforestation was used as commercial forests, the carbon storage of all China’s arbor forests also showed an increasing trend year by year, but it was slightly lower than the scenario in which all new afforestation was used as public welfare forest. Under this scenario, the average annual carbon sink of all arbor forests in China was 240.1 MgC/a, and the cumulative carbon stock was 10,085.9 MtC. The vegetation carbon density was lower than when new afforestation was used as public welfare forests, reaching 68.6 MgC/ha by 2060.

### 3.3. Carbon Emission Mitigation of Forestry Bioenergy

Deforested fallen material and waste forest products can be used as forestry bioenergy. These bioenergies can be used as a substitute for coal, thereby reducing carbon emissions. Since there was no deforestation setting for public welfare forests, this paper accounted for the emission reduction by replacing coal with bioenergy generated from commercial forests. The commercial forests contained two parts, with one being existing commercial forests and the other newly planted commercial forests.

**Figure 5:**
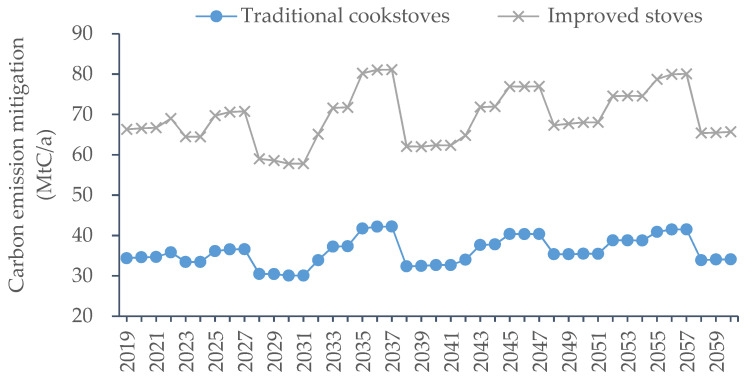
Carbon emission mitigation by forestry bioenergy of existing commercial forest.

For the existing commercial forest, the emission reductions from two biomass combustion technologies, traditional cookstoves and improved stoves, are shown in Figure 5. Although the annual carbon sink of the existing commercial forest showed a downward trend during the simulation period, the emission reduction effect of bioenergy substitution for coal was significant. Affected by periodic deforestation, the mitigated carbon emissions brought about by the substitution of bioenergy for coal also fluctuated periodically. From 2018 to 2060, using traditional cookstoves to burn bioenergy reduced greenhouse gas emissions by 1516.0 MtC cumulatively, with an average annual reduction of 36.1 MtC/a. The improvement in energy use efficiency resulted in more dramatic emission mitigation. As can be seen in Figure 5, the improved stoves were much more effective than traditional cookstoves, with a cumulative reduction of 2911.2 MtC and an average annual reduction of 69.3 MtC/a.

Without considering the carbon reduction effect of forestry bioenergy, the existing arbor forests in China accumulated carbon sequestration of 7171.5 MtC, with an average annual carbon sink of 170.8 MtC/a. If carbon reduction from bioenergy was included in the scope of the carbon sequestration of arbor forests vegetation, when traditional cookstoves of bioenergy were adopted, the cumulative carbon sequestration increased to 8687.6 MtC, with an average annual carbon sink of 206.8 MtC/a, which is a 21.1% increase in annual carbon sink capacity compared to the forest carbon sink when bioenergy was not included. The reduction effect of using improved stoves was even more remarkable, with a cumulative sequestration of 10,082.7 MtC and average annual sequestration of 240.1 MtC/a, which is an increase of 40.6% in annual carbon sink capacity compared to the forest carbon sink excluding bioenergy. It can be seen that for existing commercial forests, the reuse of their litter and waste wood products should be promoted, and these forestry bioenergies could be extremely helpful to the forestry sector in enhancing carbon sinks.

**Figure 6:**
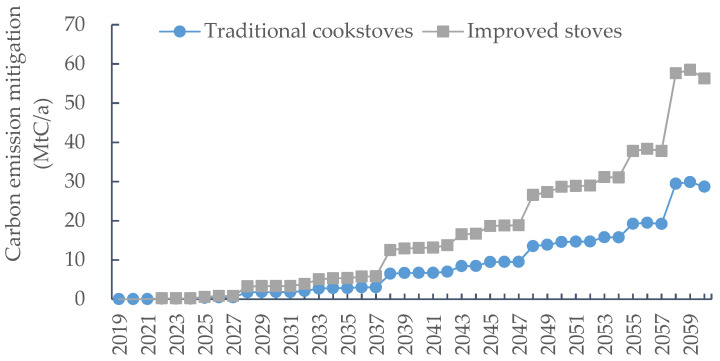
Carbon emission mitigation by forestry bioenergy of new planted commercial forest.

Figure 6 reveals that there was a gradual rise in the mitigated carbon emission of bioenergy from newly planted commercial forests. When all the new afforestation was used as commercial forests, the emission reduction of bioenergy was not significant in the early stage of the simulation. This is partly because the newly planted forests were not yet mature and would not be deforested, so there was relatively little fallen material converted to bioenergy; on the other hand, since the area of newly planted forests was very limited at the beginning, the forestry bioenergy that could be obtained was little. The emission reduction of new plantations was dramatically enhanced when they matured. The use of traditional cookstoves, with bioenergy replacing coal, reduced greenhouse gas emissions by 28.7 MtC/a in 2060. During the simulation period, the average annual emission reduction was 8.4 MtC/a, and the cumulative carbon emission reduction was 352.3 MtC. The emission reduction in terms of improved stoves was the most significant, with a carbon emission reduction of 56.3 MtC/a in 2060. From 2019 to 2060, the average annual emission reduction was 16.4 MtC/a, and the cumulative carbon emission reduction was 690.3 MtC, which was double that of traditional cookstoves.

Combining the carbon emission reduction of bioenergy with the carbon sink of newly planted commercial forest, the scale of emission reduction from newly planted commercial forests was significantly improved. When traditional cookstoves were adopted, newly planted commercial forests sequestered a total of 3266.7 MtC during the simulation period, with an average annual carbon sink of 144.8 MtC/a, an increase of 12.2% compared to that when biomass was not included. When improved stoves were adopted, newly planted commercial forests sequestered a total carbon of 3604.7 MtC, with an average annual carbon sink of 161.0 MtC/a, an increase of 24.8% compared to that without bioenergy. From the results of the above analysis, it can be concluded that the use of bioenergy contributes significantly to carbon emission reduction, especially when bioenergy combustion technology is promoted. However, the comparison of all simulation results showed that the average annual carbon sink of newly planted public welfare forests was 162.7 MtC/a, which was slightly higher than that when improved stoves were used. This indicates that with a limited improvement in bioenergy utilization efficiency, the carbon sink of newly planted commercial forests was still lower than that of newly planted public welfare forests, even considering the carbon reduction effect of bioenergy. Therefore, in the context of China’s strengthening of ecological civilization construction, from the perspective of reducing emissions, China’s new afforestation should be dominated by ecological public welfare forests, which have more significant advantages in reducing emissions and ecological services.

Combining the simulation results in Section 3.2 and Section 3.3, it can be found that in order to enhance the carbon sequestration capacity of China’s arbor forest ecosystem, the use of bioenergy should be further enhanced in commercial forests in China’s existing arbor forests by adopting improved stoves, and new plantations should for the most part be ecological public welfare forests. Under this scenario, the cumulative carbon sequestration of China’s arbor forest vegetation from 2018 to 2060 would reach 13,785.5 MtC, with an average annual carbon sink of 328.2 MtC/a. The study by Wu et al. showed that the annual average carbon emissions from energy consumption in China from 2018 to 2060 would be 3592.3 MtC/a [53]. This means that the annual carbon sink of arbor forest vegetation would be about 9.1% of the energy consumption carbon emissions. In summary, against the background that China is the leading carbon emitter in the world, the carbon sequestration of China’s forest ecosystems needs to be further improved in order to achieve the “Carbon Peaking and Carbon Neutrality” target, and various carbon reduction and sequestration measures should be implemented in concert.

## 4. Discussion

### 4.1. Comparison with Other Studies’ Findings

This article simulated the vegetation carbon sequestration of Chinese arbor forest from 2018 to 2060 with the CO2FIX model. To validate the simulation results, we compared and analyzed the simulation results of forest carbon sinks in this paper and other studies. Sun and Liu, Zhao et al., Tang et al., and others have analyzed carbon sequestration by forest vegetation in China over historical periods [15,16,18]. Zhao et al. studied forest carbon sinks from 1977 to 2013 based on China’s seven forest inventory data. The results showed that biomass carbon stocks reached 7.27 PgC by 2013 and the biomass carbon density of forest increased from 38.18 MgC/ha to 44.52 MgC/ha during the study period [16]. Sun and Liu concluded that the carbon content of forest vegetation in China is 8.65 ± 1.52 PgC (after 2007) and the biomass carbon density is approximately 43.11 ± 10.42 MgC/ha [15]. Tang et al. indicated that the forest biomass carbon density in China from 2010 to 2015 was 55.7 ± 9.1 MgC/ha. The simulation results in this paper indicated that the vegetation carbon density of China’s arbor forest in 2018 was 40.8 MgC/ha, which is closer to the simulation results of the above studies. The Chinese forest inventory data shows that the total carbon stock of China’s forest vegetation was 8427 MtC (8.4 PgC) in 2013, increasing to 9186 MtC (9.2 PgC) by 2018, with an average annual increase of 151.8 MtC/a [18]. In this paper, the vegetation carbon stock of China’s arbor forest reached 7344.8 MtC (7.3 PgC) in 2018, which was slightly lower than the data from China’s forest resources inventory. This is because in addition to arbor forest, China’s forest inventory data still includes carbon stocks of other forest types. The average annual carbon sink of existing arbor forest vegetation in China from 2019 to 2060 was 170.8 MtC/a, which was greater than the annual carbon sink inferred from the forest inventory, with this being influenced by the expansion of forest areas and growth in terms of tree age.

Yao et al. and Qiu et al. predicted China’s forest carbon sink, respectively [20,23]. Yao et al. estimate China’s forest biomass C sequestration to be 6.69 PgC from the 2000s to the 2040s, with an average annual increase of 0.17 PgC/a. The total forest biomass in China would increase by 8.89–10.37 PgC by the end of 2040s [20]. Qiu et al. suggested that the forest biomass carbon stocks in 2020 would be 9233.9 MtC (9.2 PgC), with a carbon intensity of 52.8 MgC/ha, while the carbon stock in 2050 will be 13,901.76 MtC (13.9 PgC), with a carbon intensity of 70.43 MgC/ha [23]. In this study, the vegetation carbon stock of China’s arbor forest for the years 2020 and 2050 were 7894.4 MtC (7.9 PgC) and 15,509.9 MtC (15.5 PgC), respectively, which are greater than the simulated results of Qiu et al. [23]. This is due to the larger forest area in this study. By 2050, the forest area of this study was 254.2 Mha, which is larger than the 197.4 Mha mentioned in Qiu et al. [23].

Although forestry bioenergy can play an important role in mitigating carbon emissions, there are few quantitative studies on forestry bioenergy of China. Moreover, their findings vary widely and are influenced by various factors such as the definition of forestry residue, modelling methods, and energy use efficiency [54]. Kang et al. pointed out that the total potential of domestic bioenergy in 2016 was the equivalent to 27.6% of China’s energy consumption. If this potential can be realized in a planned way to displace fossil fuels during the period 2020–2050, cumulative greenhouse gas emissions mitigation would be in the range of 450–1598.1 MtC [28]. In addition to the analysis of forest carbon sequestration, this paper also estimated carbon reduction from forestry bioenergy. The simulation results in this paper showed that from 2020 to 2050, the use of traditional cookstoves to replace coal with forestry bioenergy could reduce carbon emissions by 1103.9 MtC, while improved stoves had a more significant reduction of 2117.8 MtC. These simulation results were significantly higher than the results of Kang et al. [28], because this paper calculated the emission reduction potential of forest bioenergy under ideal conditions and adopted a more efficient bioenergy utilization technology. In summary, although the simulation results of this paper differed from those of other studies, the simulation results were generally similar. Moreover, the quantitative modelling of carbon reduction from forestry bioenergy in this paper provided data support for the forestry sector to cope with climate change.

### 4.2. Uncertainty

Studies on the carbon sink of China’s forests have been widely conducted, and the differences in estimation methods, databases, and estimation scopes directly bring about uncertainties in the estimation results. In this paper, there are certain uncertainties in the estimation of carbon sequestration by arbor forest vegetation and carbon reduction by biomass energy with regard to the following:

- There is geographical variability in the growth of arbor forests. Although the parameters of the CO2FIX model had been localized in this paper, the influence of geographical variability on the estimation results cannot be avoided.
- The allocation of new afforestation areas. Variations in vegetation carbon density among different tree species lead to differences in the estimation results under different area allocation schemes.
- The efficiency of bioenergy utilization. Although the actual energy consumption of rural China was considered, this paper set out to use bioenergy as a substitute for coal; however, this was an ideal situation and would deviate to some extent from the actual situation.
- The models used for estimating carbon sinks. The CO2FIX model simulates the forest carbon cycle without considering the disturbances in forest carbon sinks caused by climate change, increased atmospheric CO_2_ concentrations, and fires, etc. This may also bring uncertainty to the results of this study.

Overall, these above uncertainties would directly lead to differences between the results of this paper and others, so follow-up work should be done to further improve this study.

## 5. Conclusions

Based on the ninth China forest resources inventory data and the CO2FIX model, this paper explored the carbon sink of China’s arbor forest vegetation from 2018 to 2060 and accounted for carbon emission mitigation in terms of forestry bioenergy as a substitute for coal. From the perspective of tree species, the vegetation carbon sink of China’s forests mainly comes from broad-leaved mixed forest, quercus, Chinese fir, larch, and soft broad-leaved forest. There was a steady increase in the vegetation carbon storage of China’s existing arbor forests, reaching 14,516.4 MtC by 2060, which was dominated by the carbon stock of public welfare forests. The annual carbon sink of existing arbor forests generally showed a fluctuating downward trend. If the afforestation commenced in 2019, China’s forest coverage rate would reach 30.7% by 2050. When all newly planted forests were used as public welfare forests, the accumulated carbon sink amounted to 3702.8 MtC, which was higher than that of newly planted commercial forests. The vegetation carbon storage of total arbor forest in China rose annually from 2018 to 2060, with the annual carbon sink as a whole fluctuating around 250 MtC/a.

Given that arbor forests can act as both a carbon sink and a source of bioenergy, this paper accounted for the carbon reduction brought about by replacing coal with forestry bioenergy. For existing commercial forests, the emission reduction in terms of forestry bioenergy as a substitute for fossil energy was significant. The average annual emission reduction reached 36.1 MtC/a with traditional cookstoves and even more so with improved stoves, reaching 69.3 MtC/a. Therefore, for existing forests, especially existing commercial forests, bioenergy utilization technology should be improved to strengthen its substitution effect when replacing fossil energy and further promote carbon emission reduction. For new afforestation, China’s new afforestation should primarily be ecological public welfare forests, which exhibited more significant advantages in terms of emission reduction. Combining carbon sequestration from forests and carbon reduction from forestry bioenergy, the vegetation carbon sequestration of the total arbor forest in China was remarkable if all new plantations were used as public welfare forests, with the average annual carbon sink being about 9.1% of China’s carbon emissions from energy consumption. Nevertheless, the carbon sequestration capacity of China’s forest ecosystems needs to be further improved to achieve the “Carbon Peaking and Carbon Neutrality” target, and the coordinated implementation of various carbon reduction and sequestration measures is required.

## Figures and Tables

**Table 1:** Volume and area of dominant tree species of Chinese arbor forests in 2018.

Tree Species	Volume (Mm^3^)	Area (Mha)		
		Public Welfare Forest	Commercial Forest	Total
Broad-leaved mixed forest	4390.4	24.6	19.9	44.5
Quercus	1386.6	9.9	5.4	15.3
Fir	1325.0	2.9	0.7	3.6
Coniferous and broad-leaved mixed forest	1241.5	7.6	6.6	14.2
Larch	1123.0	7.7	3.2	10.8
Spruce	972.7	3.7	0.7	4.4
Birch	922.9	7.5	2.9	10.4
Chinese fir	852.0	4.9	6.5	11.4
Masson pine	626.1	3.7	4.3	8.0
Poplar	612.4	5.2	3.1	8.3
Coniferous mixed forests	574.7	3.0	2.8	5.8
*Pinus yunnanensis*	501.0	2.4	1.9	4.3
*Pinus densata*	332.4	1.2	0.3	1.5
Soft broad-leaved forest	302.6	2.8	1.5	4.3
Cypress	232.0	2.5	1.2	3.7
*Eucalyptus*	215.6	1.8	3.6	5.5
Hardwood broad-leaved forest	154.0	1.7	1.1	2.8
*Pinus tabulaeformis*	145.1	1.8	0.6	2.5
*Pinus kesiya*	61.6	0.3	0.3	0.6

Note data sources [47].

**Table 2:** The carbon content of different organs of tree species.

Tree Species	Stem (%)	Root (%)	Foliage (%)	Branch (%)
Quercus	44.34	42.26	45.86	44.73
Spruce, Fir	47.54	48.54	50.67	49.35
Coniferous and broad-leaved mixed forest	48.12	48.03	47.22	47.6
Larch	47.52	47.02	47.09	47.77
Birch	45.61	45.19	47.55	47.98
Chinese fir	47.71	43.17	47.95	46.52
Masson pine	47.91	45.96	49.8	48.32
Poplar	46.09	43.76	44.8	46.13
*Pinus kesiya, Pinus densata*, Coniferous mixed forests	47.32	47.27	49.28	48.78
*Pinus yunnanensis*	47.91	45.96	49.8	48.32
Soft broad-leaved forest	45.21	43.5	44.55	44.38
Cypress	48.33	46.13	50.53	48.31
*Eucalyptus*	45.5	45.23	46.18	45.3
Broad-leaved mixed forest, Hardwood broad-leaved forest	45.36	43.68	45.61	45.39
*Pinus tabulaeformis*	47.36	45.92	49.68	48.3

Note data sources [48].

**Table 3:** Greenhouse gas emission factors.

Fuel	Technology	Emission Factors (g/kg)					References
		CO_2_	CH_4_	N_2_O	CO	TNMOC	
Coal	Anthracite stoves	2876.26	8.78	0.04	58.49	5.82	[52]
Forestry bioenergy	Traditional cookstoves	0	9.4	0.08	64.7	9.65	[29]
	Improved stoves	0	7.92	0.06	69.5	6.84	

**Table 4:** Cumulative carbon stock by dominant tree species of China’s existing arbor forests from 2019 to 2060 (MtC).

Tree Species	Public Welfare Forest	Commercial Forest	Total
Broad-leaved mixed forest	2012.7	433	2445.8
Quercus	357.9	113.4	471.4
Fir	55.5	−3.9	51.6
Coniferous and broad-leaved mixed forest	163.4	67	230.5
Larch	348.2	46.4	394.5
Spruce	87.5	4.6	92.1
Birch	38	43.6	81.5
Chinese fir	341.4	163.5	504.8
Masson pine	203.3	102.2	305.5
Poplar	330.2	43	373.2
Coniferous mixed forests	44.9	28.5	73.4
*Pinus yunnanensis*	27.2	10	37.2
*Pinus densata*	38.8	2.4	41.2
Soft broad-leaved forest	403.5	48.5	452
Cypress	272	72.5	344.5
*Eucalyptus*	93.5	244.9	338.4
Hardwood broad-leaved forest	346.2	67.4	413.6
*Pinus tabulaeformis*	48.8	8.6	57.5
*Pinus kesiya*	2.6	3.5	6.1

**Table 5:** Vegetation carbon sink by total arbor forests in China.

Scenario	Category	2018	2020	2030	2040	2050	2060
China’s total arbor forests (new afforestation for public welfare forests)	Area (Mha)	179.9	183.3	200.5	224.1	254.2	254.2
	Vegetation carbon stock (MtC)	7344.8	7894.4	10,480.9	12,871.4	15,510.0	18,219.1
	Vegetation carbon sink (MtC/a)	—	274.5	249.4	268.3	272.3	279.4
	Vegetation carbon density (MgC/ha)	40.8	43.1	52.3	57.4	61.0	71.7
China’s total arbor forests (new afforestation for commercial forests)	Area (Mha)	179.9	183.3	200.5	224.1	254.2	254.2
	Vegetation carbon stock (MtC)	7344.8	7894.4	10,463.3	12,757.3	15,169.9	17,430.7
	Vegetation carbon sink (MtC/a)	—	274.5	244.2	251.0	241.4	211.1
	Vegetation carbon density (MgC/ha)	40.8	43.1	52.2	56.9	59.7	68.6

## Data Availability

Not applicable.

## References

[1] Wei Y.M., Chen K.Y., Kang J.N., Chen W.M., Wang X.Y., Zhang X.Y. (2022). Policy and management of carbon peaking and carbon neutrality: A literature review. Engineering.

[2] Cai L.Y., Luo J., Wang M.H., Guo J.F., Duan J.L., Li J.T., Li S., Liu L.T., Ren D.P. (2022). Pathways for municipalities to achieve carbon emission peak and carbon neutrality: A study based on the leap model. Energy.

[3] Cai B.F., Zhang L., Lei Y., Wang J.N. (2022). A deeper understanding of the CO_2_ emission pathway under China’s carbon emission peak and carbon neutrality goals. Engineering.

[4] Qi J.J., Dauvergne P. (2022). China and the global politics of nature-based solutions. Environ. Sci. Policy.

[5] Gómez Martín E., Máñez Costa M., Egerer S., Schneider U.A. (2021). Assessing the long-term effectiveness of nature-based solutions under different climate change scenarios. Sci. Total Environ..

[6] Zhang X.X., Wu L.Y., Ma X.Z., Qin Y.C. (2022). Dynamic computable general equilibrium simulation of agricultural greenhouse gas emissions in China. J. Clean. Prod..

[7] Chu X., Zhan J.Y., Li Z.H., Zhang F., Qi W. (2019). Assessment on forest carbon sequestration in the Three-North Shelterbelt program region, China. J. Clean. Prod..

[8] Li W., Zhang S.H., Lu C. (2022). Exploration of China’s net CO_2_ emissions evolutionary pathways by 2060 in the context of carbon neutrality. Sci. Total Environ..

[9] Lin B.Q., Ge J.M. (2019). Carbon sinks and output of China’s forestry sector: An ecological economic development perspective. Sci. Total Environ..

[10] Song Z.L., Liu H.Y., Strömberg C.A., Wang H.L., Strong P.J., Yang X.M., Wu Y.T. (2018). Contribution of forests to the carbon sink via biologically-mediated silicate weathering: A case study of China. Sci. Total Environ..

[11] Zhang P.Y., He J.J., Hong X., Zhang W., Qin C.Z., Pang B., Li Y.Y., Liu Y. (2018). Carbon sources/sinks analysis of land use changes in China based on data envelopment analysis. J. Clean. Prod..

[12] Machado R.R., Conceição S.V., Leite H.G., Souza A.L.D., Wolff E. (2015). Evaluation of forest growth and carbon stock in forestry projects by system dynamics. J. Clean. Prod..

[13] Rosa C.M.D., Marques M.C. (2022). How are biodiversity and carbon stock recovered during tropical forest restoration? Supporting the ecological paradigms and political context involved. J. Nat. Conserv..

[14] Yu G.R., Zhu J.X., Xu L., He N.P. (2022). Technological approaches to enhance ecosystem carbon sink in China: Nature-based solutions. Bull. Chin. Acad. Sci..

[15] Sun W.L., Liu X.H. (2020). Review on carbon storage estimation of forest ecosystem and applications in China. For. Ecosyst..

[16] Zhao M.M., Yang J.L., Zhao N., Xiao X.M., Yue T.X., Wilson J.P. (2021). Estimation of the relative contributions of forest areal expansion and growth to China’s forest stand biomass carbon sequestration from 1977 to 2018. J. Environ. Manag..

[17] Ma X.Z., Wang Z. (2011). Estimation of provincial forest carbon sink capacities in chinese mainland. Chin. Sci. Bull..

[18] Tang X.L., Zhao X., Bai Y.F., Tang W.T., Zhao Y.C., Wan H.W., Xie Z.Q., Shi X.A., Wu B.F., Wang G.X. (2018). Carbon pools in China’s terrestrial ecosystems new estimates based on an intensive field survey. Proc. Natl. Acad. Sci. USA.

[19] Stephenson N.L., Das A.J., Condit R., Russo S.E., Baker P.J., Beckman N.G., Coomes D.A., Lines E.R., Morris W.K., Rüger N. (2014). Rate of tree carbon accumulation increases continuously with tree size. Nature.

[20] Yao Y.T., Piao S.L., Wang T. (2018). Future biomass carbon sequestration capacity of Chinese forests. Sci. Bull..

[21] Zhao M.M., Yang J.L., Zhao N., Liu L., Du L., Xiao X.M., Yue T.X., Wilson J.P. (2021). Spatially explicit changes in forest biomass carbon of China over the past 4 decades: Coupling long-term inventory and remote sensing data. J. Clean. Prod..

[22] Fang J.Y., Chen A.P., Peng C.H., Zhao S.Q., Ci L.J. (2001). Changes in forest biomass carbon storage in China between 1949 and 1998. Science.

[23] Qiu Z.X., Feng Z.K., Song Y.N., Li M.L., Zhang P.P. (2020). Carbon sequestration potential of forest vegetation in China from 2003 to 2050: Predicting forest vegetation growth based on climate and the environment. J. Clean. Prod..

[24] Lin B.Q., Ge J.M. (2019). Valued forest carbon sinks: How much emissions abatement costs could be reduced in China. J. Clean. Prod..

[25] National Forestry and Grassland Administration http://www.gov.cn/xinwen/2016-07/28/content_5095504.htm.

[26] Daigneault A., Favero A. (2021). Global forest management, carbon sequestration and bioenergy supply under alternative shared socioeconomic pathways. Land Use Policy.

[27] Saez de bikuña K., Garcia R., Dias A.C., Freire F. (2020). Global warming implications from increased forest biomass utilization for bioenergy in a supply-constrained context. J. Environ. Manag..

[28] Kang Y.T., Yang Q., Bartocci P., Wei H.J., Liu S.S., Wu Z.J., Zhou H.W., Yang H.P., Fantozzi F., Chen H.P. (2020). Bioenergy in China: Evaluation of domestic biomass resources and the associated greenhouse gas mitigation potentials. Renew. Sustain. Energy Rev..

[29] Schelhaas M.J., Van Esch P.W., Groen T.A., De Jong B.H.J., Kanninen M., Liski J., Masera O., Mohren G.M.J., Nabuurs G.J., Palosuo T. (2004). CO2FIX V 3.1-A Modelling Framework for Quantifying Carbon Sequestration in Forest Ecosystems.

[30] Repo A., Ahtikoski A., Liski J. (2015). Cost of turning forest residue bioenergy to carbon neutral. For. Policy Econ..

[31] Righelato R., Sprackle D.V. (2007). Carbon mitigation by biofuels or by saving and restoring forests. Science.

[32] Yang J., Dai G.H., Ma L.Y., Jia L.M., Wu J., Wang X.H. (2013). Forest-based bioenergy in China: Status, opportunities, and challenges. Renew. Sustain. Energy Rev..

[33] Qin Z.C., Zhuang Q.L., Cai X.M., He Y.J., Huang Y., Jiang D., Lin E., Liu Y.L., Tang Y., Wang Q.M. (2018). Biomass and biofuels in China: Toward bioenergy resource potentials and their impacts on the environment. Renew. Sustain. Energy Rev..

[34] Knápek J., Králík T., Vávrová K., Valentová M., Horák M., Outrata D. (2021). Policy implications of competition between conventional and energy crops. Renew. Sustain. Energy Rev..

[35] Fu T.C., Ke J.H., Zhou S.K., Xie G.H. (2020). Estimation of the quantity and availability of forestry residue for bioenergy production in China. Resour. Conserv. Recycl..

[36] Lin B.Q., Ge J.M. (2020). To harvest or not to harvest? forest management as a trade-off between bioenergy production and carbon sink. J. Clean. Prod..

[37] Daigneault A., Baker J.S., Guo J., Lauri P., Favero A., Forsell N., Johnston C., Ohrel S.B., Sohngen B. (2022). How the future of the global forest sink depends on timber demand, forest management, and carbon policies. Glob. Environ. Change.

[38] Zhao J.F., Liu D.S., Cao Y., Zhang L.J., Peng H.W., Wang K.L., Xie H.F., Wang C.Z. (2022). An integrated remote sensing and model approach for assessing forest carbon fluxes in China. Sci. Total Environ..

[39] Rizvi R.H., Newaj R., Prasad R., Handa A.K., Alam B., Chavan S.B., Saxena A., Karmakar P., Jain A.K., Chaturvedi M. (2016). Assessment of carbon storage potential and area under agroforestry systems in Gujarat Plains by CO2FIX model and remote sensing techniques. Curr. Sci..

[40] Nepal P., Ince P.J., Skog K.E., Chang S.J. (2012). Projection of U.S. forest sector carbon sequestration under U.S. and global timber market and wood energy consumption scenarios, 2010–2060. Biomass Bioenergy.

[41] Wang W.F., Duan Y.X., Zhang L.X., Wang B., Li X.J. (2016). Review on forest carbon sequestration counting methodology under global climate change. J. Nanjing For. Univ. (Nat. Sci. Ed.).

[42] Negash M., Kanninen M. (2015). Modeling biomass and soil carbon sequestration of indigenous agroforestry systems using CO2FIX approach. Agric. Ecosyst. Environ..

[43] Kaonga M.L., Bayliss-Smith T.P. (2012). Simulation of carbon pool changes in woodlots in eastern Zambia using the CO2FIX model. Agrofor. Syst..

[44] Bordoloi R., Das B., Tripathi O., Sahoo U., Nath A., Deb S., Das D., Gupta A., Devi N., Charturvedi S. (2021). Satellite based integrated approaches to modelling spatial carbon stock and carbon sequestration potential of different land uses of Northeast India. Environ. Sustain. Indic..

[45] Röder M., Thiffault E., Martínez-Alonso C., Senez-Gagnon F., Paradis L., Thornley P. (2019). Understanding the timing and variation of greenhouse gas emissions of forest bioenergy systems. Biomass Bioenergy.

[46] Tang W.G., Zhang S.H. (2018). Optimal decision model and solution for carbon sequestration by afforestation. Comput. Math. Appl..

[47] National Forestry and Grassland Administration (2019). China Forest Resources Report 2014–2018.

[48] Wang W.T., Tang X.L., Huang M. (2018). Carbon Storage in China’s Forest Ecosystems-Dynamics and Mechanisms.

[49] National Public Service Platform for Standards Information. https://std.samr.gov.cn/hb/search/stdHBDetailed?id=8B1827F2424CBB19E05397BE0A0AB44A.

[50] Wang S.X., Wei W., Du L., Li G.H., Hao J.M. (2009). Characteristics of gaseous pollutants from biofuel-stoves in rural China. Atmos. Environ..

[51] Deng M.S., Li P.C., Ma R.J., Shan M., Yang X.D. (2020). Air pollutant emission factors of solid fuel stoves and estimated emission amounts in rural Beijing. Environ. Int..

[52] IPCC https://www.ipcc-nggip.iges.or.jp/EFDB/main.php.

[53] Wu L.Y., Liu C.X., Ma X.Z., Liu G.B., Miao C.H., Wang Z. (2019). Global carbon reduction and economic growth under autonomous economies. J. Clean. Prod..

[54] Baker J.S., Wade C.M., Sohngen B.L., Ohrel S., Fawcett A.A. (2019). Potential complementarity between forest carbon sequestration incentives and biomass energy expansion. Energy Policy.

